# Functional dissociation along the rostrocaudal axis of Japanese quail hippocampus

**DOI:** 10.1371/journal.pone.0277414

**Published:** 2022-11-14

**Authors:** Chelsey C. Damphousse, Noam Miller, Diano F. Marrone

**Affiliations:** Dept. of Psychology, Wilfrid Laurier University, Waterloo, ON, Canada; Pennsylvania State University, UNITED STATES

## Abstract

The mammalian hippocampus (Hp) can be functionally segregated along its septotemporal axis, with involvement of dorsal hippocampus (dHp) in spatial memory and ventral hippocampus (vHp) in stress responses and emotional behaviour. In the present study, we investigate comparable functional segregation in proposed homologues within the avian brain. Using Japanese quail (Coturnix Japonica), we report that bilateral lesions of the rostral hippocampus (rHp) produce robust deficits in a spatial Y-maze discrimination (YMD) test while sparing performance during contextual fear conditioning (CFC), comparable to results from lesions to homologous regions in mammals. In contrast, caudal hippocampus (cHp) lesions failed to produce deficits in either CFC or YMD, suggesting that, unlike mammals, both cHp and rHp of birds can support emotional behavior. These observations demonstrate functional segregation along the rostrocaudal axis of the avian Hp that is comparable in part to distinctions seen along the mammalian hippocampal septotemporal axis.

## Introduction

The hippocampus (Hp) is a structure critical to many forms of memory and spatial navigation across a number of species. Given the many computations this structure must complete in order to serve these complex cognitive functions, it is not surprising that the Hp is not a unitary structure but is instead segregated into multiple functionally distinct subregions. One important functional distinction is along the dorsoventral axis, also referred to as the septotemporal, or “long” axis. Evidence of functional distinctions along this axis have been noted since the earliest studies examining the behavioral effects of Hp lesions [1–3], and numerous studies have confirmed and extended these observations (reviewed in [4]). While the precise nature of the functional domains within the Hp remains a topic of debate [4–6], there is consensus that the dorsal Hp (dHp), for instance, is critical for spatial memory in small environments [7, 8] while the ventral region (vHp) is more critical to emotional behavior and stress responses including contextual fear [9–12]. One way to consider this dissociation is as a difference in resolution. Lesions of the dHp severely impair the performance of tasks that require high-resolution recollection of the precise spatial locations of rewards or objects, such as finding baited arms in a radial arm maze [13–15] or the location of a hidden platform in a water maze [7, 8], or recognizing and approaching an object that is in a novel spatial location [16] while lesions to vHp do not affect these behaviors. In contrast, tasks that require pairing an event with a context in general terms, such as contextual fear conditioning (CFC), are impacted by pre-training lesions to vHp [17, 18] and not dHp [19–21].

The avian Hp is a proposed homologue of the mammalian Hp for numerous reasons including similarities in development, connectivity and neurotransmitters, and because of its role in spatial cognition (see [22–25] for review). Similar to functional gradients observed along the dorsoventral axis in mammals, several studies propose a comparable functional gradient along the rostrocaudal axis of the avian Hp. Studies of connectivity [26], gene expression [27, 28], and place cell characteristics [29] all suggest similarities between the rostral pole of the avian Hp (rHp) and the dorsal pole of the mammalian Hp (see [30] for review). What remains unknown is whether the caudal pole of the avian Hp (cHp) is functionally comparable to the ventral pole of the mammalian Hp and, if so, is there a functional dissociation between the rostral and caudal poles?

To address this, groups of Japanese quail (*Coturnix Japonica*) underwent selective lesions to either the rostral or caudal pole of the Hp. Subjects were then tested using a spatial Y-Maze discrimination (YMD) task, as well as CFC, as these types of information are known to tax the poles of the mammalian Hp differently.

## Materials and methods

### Subjects

Twenty-seven adult female Japanese quail (Spring Creek Quail Farms, Saint Ann’s, ON), aged approximately 3 months were used in this experiment. Plastic leg bands were placed on each quail at the time of arrival for identification. Quail were group housed with a minimum of 400cm^2^ floor area per bird in holding pens measuring 213 cm x 305 cm with a 12:12 light cycle and maintained at 20–25°C and 45–75% humidity. Bedding consisted of Teklad Aspen bedding with Teklad Tek-fresh provided as nesting material. Animals had *ad lib* access to water and Mazuri exotic game bird starter and Mazuri Game bird Breeder. Hides were provided in the form of overturned plastic storage containers with entrances cut out and paint roller trays filled with sand were provided for sand-bathing. Prior to behavioral testing, all animals were handled 15 min/day for at least 7 days. All procedures were approved by the animal care committee of Wilfrid Laurier University in accordance with the guidelines of the Canadian Council on Animal Care.

### Surgery

**Fig 1:**
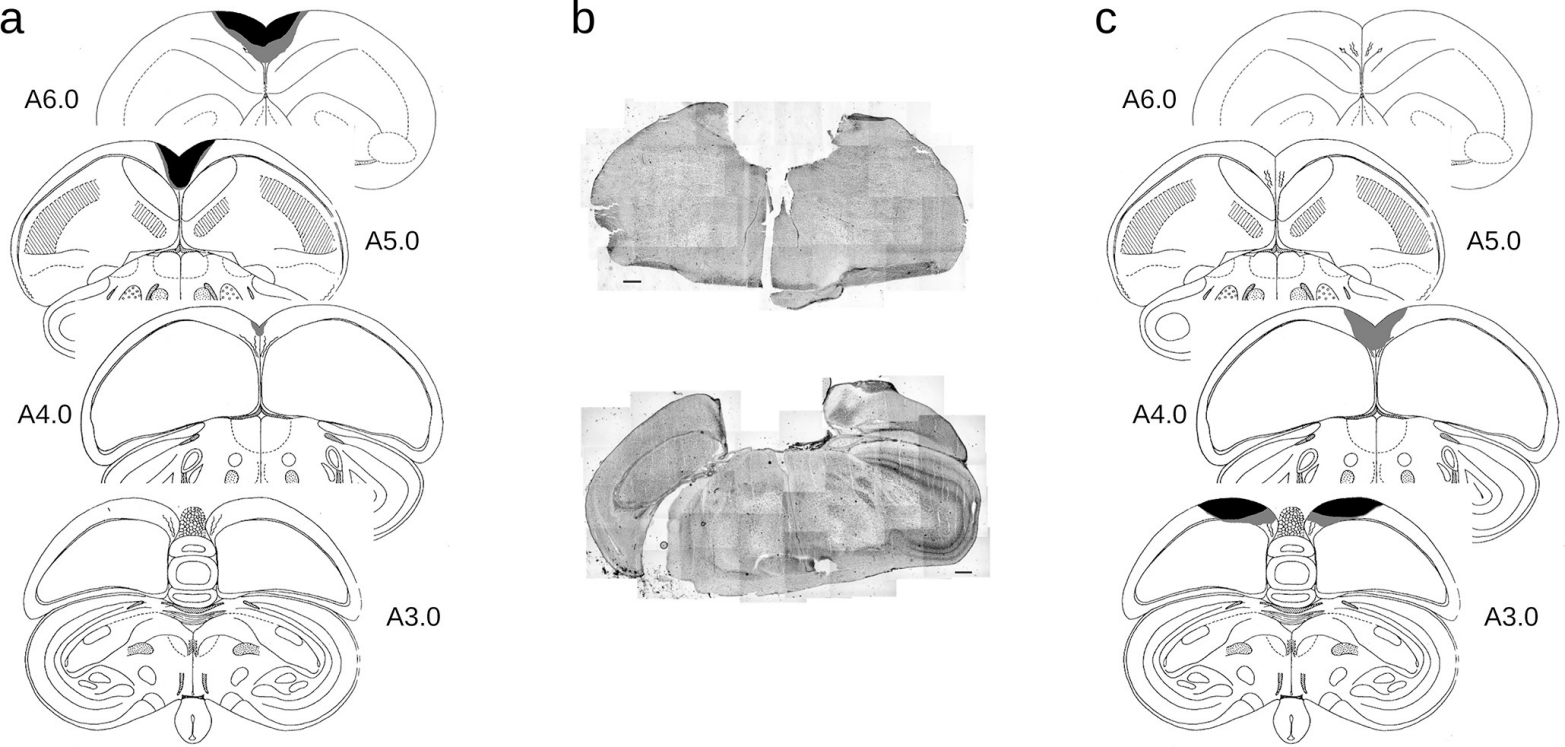
Lesion reconstruction of (a) rHp-lesioned and (c) cHp-lesioned quail included in the study. The black areas depict damage found in at least six lesioned quail. Grey areas show damage found in at least two lesioned quail. Centrally (b), tiled images of sample coronal sections (scale bar = 500 μm) are shown centrally from quail with rostral (above) and caudal (below) lesions.

**Fig 2:**
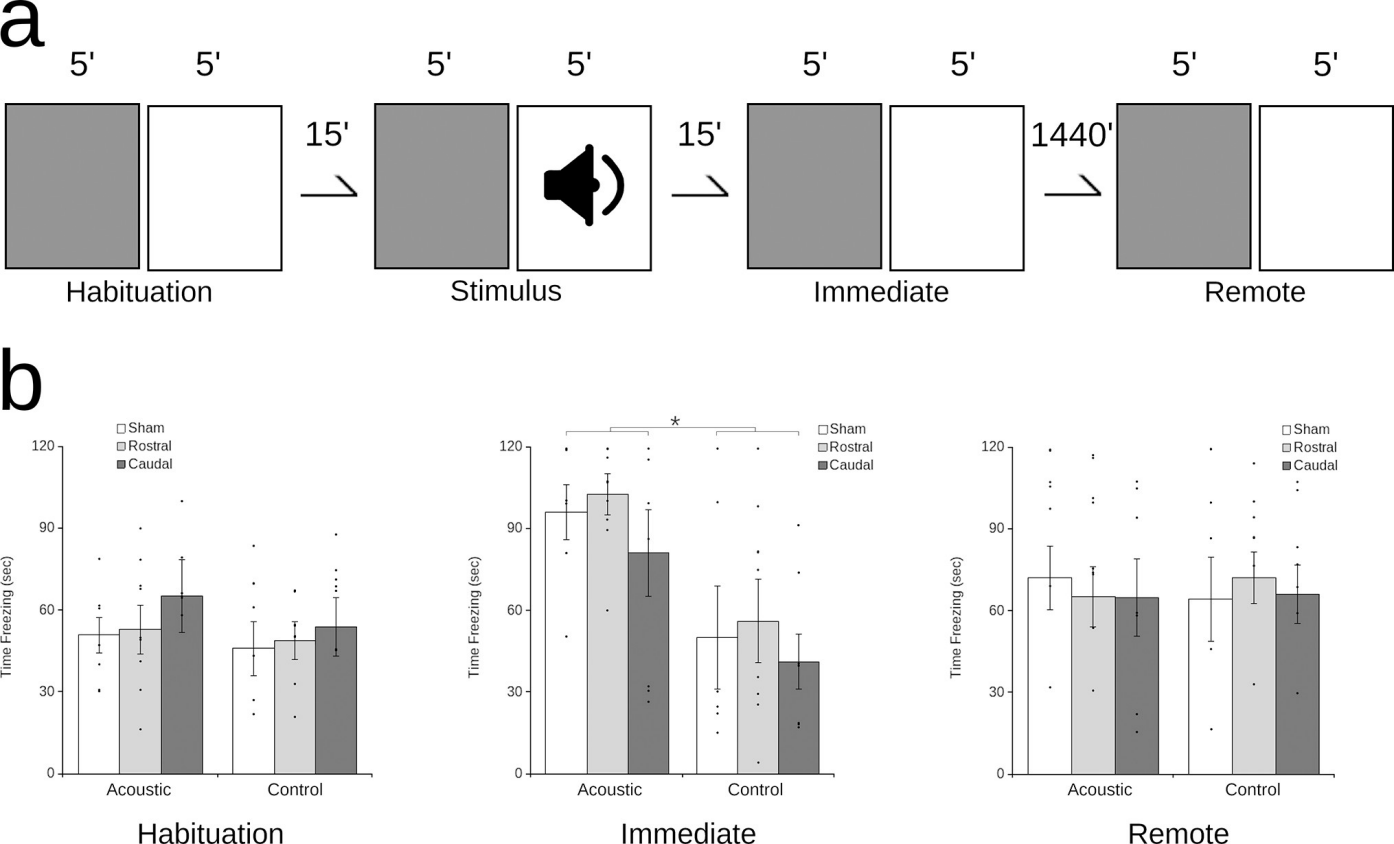
Lesions of rHp or cHp spare contextual fear conditioning (CFC). A schematic (a) shows the timing and order of CFC in context A (grey) and context B (white). Quail were pre-exposed consecutively to each context for 5 min (pre-exposure), followed by a 15 min delay. Quail were then exposed to each environment for 5 min a second time, during which they were presented with an auditory stimulus (1000 Hz, 95 dB) for 3 sec in one of the environments (stimulus). Following another 15 min delay, quail were again exposed consecutively to each context for 5 min (immediate). After 24 hours, the quail were once again exposed consecutively to each context for 5 min (remote). Analysis of the time spent freezing (b) shows that intact sham quail (white) as well as rHp-lesioned quail (light grey) and cHp-lesioned quail (dark grey) spend comparable time freezing in either environment a baseline (habituation). Following presentation of the acoustic stimulus (immediate), all quail more time freezing in the environment they received the acoustic stimulus in (acoustic) relative to the second environment (control). This difference is no longer apparent 24 hours after the presentation of the stimulus (remote) have a discrimination ratio that is not significantly different from 0, showing exploration of objects equal to random chance (bars show mean ± SEM; * = p < 0.05 significant difference between groups).

All surgeries were conducted prior to any behavioral testing. Each lesion group consisted of 9 subjects (9 rHp, 9 cHp, 9 Sham). Surgical procedures were modified from those outlined by Damphousse and colleagues [31]. Quail were anesthetized with isoflurane using a SomnoSuite anaesthesia machine (Kent Scientific, Torrington, CT) and placed in a stereotaxic instrument (Kopf Instruments, Tujunga, CA). Once the head was secured using ear bars and a nose cone, feathers were removed and the area was prepared using antibacterial cleanser (Phenrex®), 70% isopropyl alcohol, and chlorhexidine gluconate solution (Baxedin®). Following subcutaneous injection of lidocaine and epinephrine (Bimeda, Cambridge, ON) along the midline of the skull, a midline incision was made, the scalp was retracted, and a craniotomy was made over the lesion site. The Hp was removed by aspiration according to coordinates determined using a published quail brain atlas [32]. Coordinates for lesions were determined relative to where the parieto-occipital suture intersects with the midline. For rHp lesions, aspirations were 1 mm to 5 mm anterior to bregma, 1.5 mm on either side of midline, and 3 mm deep (Fig 1). Lesions to the cHp were 1 mm anterior to bregma, 3 mm posterior, 1.5 mm on either side of midline, and 3 mm deep (Fig 2). Craniotomies for sham animals were conducted at the coordinates for rHp lesions.

Craniotomies were packed using a hemostatic sponge, sealed with bone wax and the skin was sutured. After recovering on a heating pad and regaining mobility, quail were placed into individual hanging rack pigeon cages in a separate recovery room to recover for 1 week while undergoing antibiotic and analgesic treatment. Quail remained in these cages for the remainder of the experiment.

Experiments described here were run on 2 separate batches of quail. Within each batch of quail, all animals were tested on the same day in a randomized order at least 2 hours after commencement of the light cycle. Prior to beginning each day of the experiment, subjects were removed from their individual cages in the recovery room and placed into individual shoebox cages on a rack devoid of food but with ad lib access to water. Each cage was covered by a shroud and subjects were left undisturbed for 1 hour. Subjects were transported individually in their covered cages to the testing room.

In the first batch, birds were first tested in the YMD immediately following the one week recovery period (i.e., on post-surgical day 8). Following the 4-days of YMD (i.e., post-surgical days 8 through 11), quail were then tested in CFC, which occurred in one day (i.e., Day 12), followed by a remote test the following day (Day 13). In the second batch of quail, the order of training was reversed such that CFC was trained first. In both batches, quail were anaesthetized and their brains collected on post-surgical day 14.

### Contextual fear conditioning (CFC)

This experiment consisted of exposure to two visually distinct arenas in two different rooms containing unique local and distal cues, referred to as Context A and Context B (see Fig 2A). Context A consisted of a circular 90 cm diameter arena with 45 cm high walls constructed from white corrugated plastic sheeting, with a floor of the same material covered in butcher paper. Context B consisted of a square arena with 90 cm sides and 45 cm high walls constructed from painted plywood, with flooring of black haircell acrylonitrile-butadiene-styrene (ABS). Behaviour was monitored using an overhead webcam and tracking was done in real-time using ANY-maze (Stoelting, Wood Dale, IL).

The CFC procedure consisted of four phases: habituation, training, test, and remote test. Prior to beginning each day of the experiment, subjects were removed from their individual cages in the recovery room and placed into individual shoebox cages on a rack devoid of food but with ad lib access to water. Each cage was covered by a shroud and subjects were left undisturbed for 1 hour. Subjects were transported individually in their covered cages to the testing room. Habituation, training, and test all occurred on the same experimental day. During habituation, the subject was placed into Context A and allowed to explore freely for 5 min. The subject was promptly removed and the same procedure was followed in Context B with a 1 min inter-trial-interval (ITI). The subject was then placed back into a covered shoebox cage and left undisturbed for 15 minutes. During training, the subject was again exposed to Context A for 5 min, a 1 min ITI, and was then placed into Context B. After 3 min in Context B, an auditory stimulus (1000 Hz, 95 dB) was delivered for 3 sec, followed by 2 minutes of exploration. The context in which the stimulus was presented was counterbalanced across subjects. The subject was then again placed back into the covered shoebox cage and left undisturbed for 15 minutes. During test, procedures matched those in habituation with 5 min in Context A followed by 5 min in Context B. Following each phase, the arena was wiped down with 70% Ethanol to eliminate any scent cues. On the following day, subjects were given a remote test. During remote test, procedures again matched those followed during habituation with 5 min in Context A, a 1 min ITI, and 5 min in Context B.

Quail can be considered freezing when they present a characteristic crouching posture with a) total flexion of the legs and the body in contact with the floor or b) partial flexion of the legs, wide separation between feet/legs and the pectoral region in close contact with one of the walls, with eyes widely opened and accelerated respiration. Such posture, associated with the absence of other observable behaviors, has been repeatedly used to characterize freezing behavior in pigeons [33–36].

### Y-maze discrimination (YMD)

The YMD protocol used here was adapted from a previous publication testing Japanese quail [37]. Briefly, Y-maze arms measured 50 x 17 x 45 cm (L x W x H) and were constructed from clear acrylic permitting subjects to readily see distinct visual cues present on all four walls of the room. Square rod styrene tracts with removable opaque acrylic guillotine doors were installed in the two exploration arms. The floor was constructed from black haircell acrylonitrile-butadiene-styrene (ABS) and covered with wood shavings. Behaviour was monitored using an overhead webcam and tracking was done using ANY-maze (Stoelting, Wood Dale, IL).

Quail underwent three consecutive days of 10-min habituation sessions in which they had access to all arms of the maze. During the sample trial, birds were given 5 min of exploration with one arm of the maze blocked off by a guillotine door. Birds were then removed for 1 min, during which time the door was removed and the bedding in the maze was replaced to remove scent cues. Birds were then returned to the maze for a 5-min choice trial in which both arms were open. Which arm was blocked during the sample trial was counterbalanced across subjects. Note that in this spontaneous recognition memory task, no reward is provided in the maze.

### Histology

Following testing, subjects were transported to a procedure room, anesthetized with isoflurane, decapitated, and brains were extracted and flash frozen in 2-methylbutane (Sigma Aldrich, Oakville, ON). Coronal sections were cut at a thickness of 30 μm using a CM3050 cryostat (Leica) and thaw-mounted onto Superfrost Plus™ slides (Thermo Scientific, Waltham, MA). Every 6th section was then stained using Methyl Green to observe placement and extent of the lesions under a light microscope. Thaw-mounted sections were fixed for 5 min in buffered 4% formaldehyde for 5 min, rinsed in double-distilled water for 5 min, then incubated in Methyl Green for 5 minutes at room temperature. Slides were then dehydrated in a graded series of ethanol, cleared with Neo-clear, and embedded in Neo-mount (all histology reagents were obtained from Sigma Aldrich, Oakville, ON).

### Analysis

Two quail died during surgery, while another 2 were excluded for lack of movement in at least one of the 2 tests, yielding final data on 25 quail (9 rHp, 7 cHp, 7 sham).

Analysis of the CFC data was conducted using a repeated-measures analysis of variance (ANOVA) of the time spent freezing during the first 2 minutes of each trial using context (i.e., acoustically-paired vs. control) and time (i.e., immediate vs. remote) as within-subject factors and group (i.e., rHp, cHp, and sham) as between-subject factors. As an additional control, the time spent freezing in each context before any acoustical stimulation was also compared using a 2 (context) x 3 (group) ANOVA.

In the YMD, the time spent within each arm was quantified as a proportion of total exploration time. The subject was considered to be exploring an arm if their entire torso was inside the arm. The time spent exploring the novel (T_N_) and familiar (T_F_) arms (excluding the start arm) was converted into a discrimination ratio (DR) for each subject, as follows: DR = (T_N_-T_F_)/(T_N_+T_F_). These DRs were compared across groups using a one-way ANOVA.

*Post hoc* tests were conducted using Tukey’s HSD. All statistical tests were conducted using JASP [38].

## Results

### Lesions to either rostral or caudal hippocampus spare contextual fear conditioning in quail

Analysis of CFC (Fig 2) showed no significant difference between contexts (F_1,20_ = 1.03; p = 0.32) or groups (F_2,20_ = 1.42; p = 0.27) before acoustic stimulation, showing that the surgeries did not induce any baseline differences in freezing behavior. Examining the time spent freezing during the trials following stimulation failed to show a significant main effect of time (F_1,20_ = 1.03; p = 0.32) or of group (F_2,20_ = 0.33; p = 0.73). However, a significant effect of context (F_1,20_ = 7.83; p = 0.01) as well as a significant time by context interaction (F_1,20_ = 9.35; p < 0.01) were observed. This pattern of results suggests that quail across all groups in the immediate test selectively froze in the context paired with the acoustic stimulus and not in the control environment (paired vs. unpaired context: p < 0.05 for all groups), indicating that they are able to discriminate between the two contexts and retain a memory for the context in which the acoustic stimulus had been presented. Thus, it seems that the spared Hp in either lesion group support this behavior. In contrast, 24 hours later, freezing had diminished to the point at which no significant difference could be observed in any group (p > 0.05 for all groups), suggesting that the memory had degraded for all quail.

### Quail rostral hippocampus is critical for Y-maze discrimination

**Fig 3:**
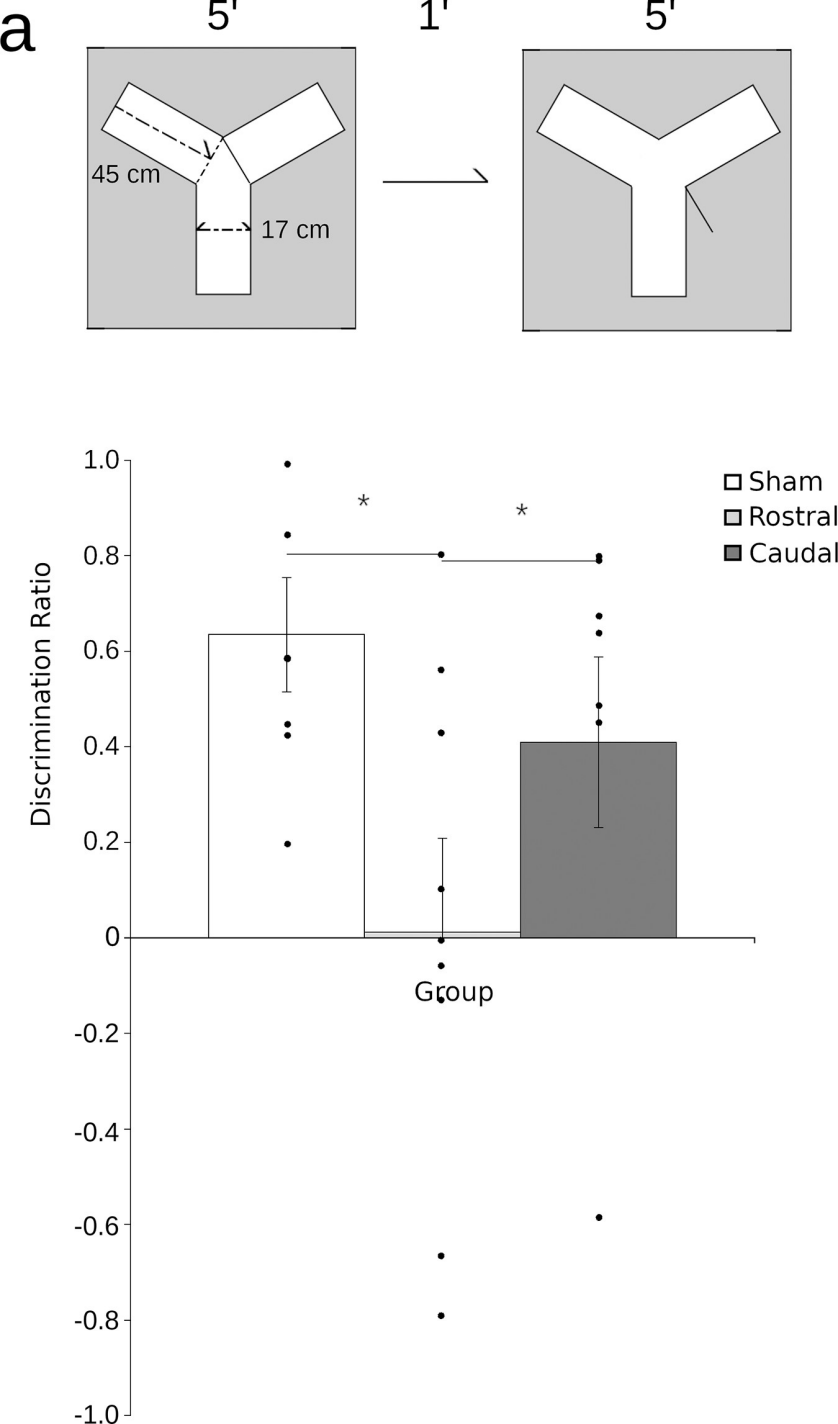
Lesions of rHp but not cHp impair Y-maze discrimination (YMD) memory. A schematic (a) demonstrates the timing of trials in YMD. Following 3 days of habituation, quail are exposed to the Y-maze for 5 min with one of the arms blocked. Quail are then removed for 1 min and the wall blocking passage to the novel arm is removed before quail are returned for another 5 min. Calculation of a discrimination ratio (b) shows that intact sham quail (white) and cHp-lesioned quail (dark grey) spend significantly more time investigating the novel arm of the maze. In contrast, quail with lesions to rHp (light grey) have a discrimination ratio that is not significantly different from 0, showing exploration of the maze arms equivalent to random chance (bars show mean ± SEM; * = p < 0.05 between groups).

Analysis of the YMD (Fig 3), yielded a significant effect of condition (F_2,20_ = 3.99; p = 0.03). Post-hoc tests showed that while rHp-lesioned quail performed significantly worse than shams (p = 0.04), cHp-lesioned quail did not (p = 0.12). This pattern of results suggests that, like mammals, the rHp of quail may disproportionately support spatial learning tasks.

## Discussion

The current results are the first to report functional segregation along the rostrocaudal axis of the avian Hp. These results partially confirm the existence of a gradient along the rostrocaudal axis that is in some ways comparable to the mammalian dorsoventral axis. In particular, we observe that the rHp is necessary for identification of spatial novelty during YMD. This observation is consistent with results produced in rodents completing comparable tasks following lesions to the dHp ([10, 39]; but see [40]). Moreover, the current results are consistent with reports of a gradient of spatial information content in avian Hp, with the greatest spatial information represented in principal cells of the rHp [29]. This pattern, which mirrors the change in information content observed along the rodent dorsoventral axis [41] further bolsters the body of evidence demonstrating that the most rostral extent of the Hp disproportionately supports high-resolution spatial information processing across both Aves and Mammalia.

The current data replicate previous observations [37] that, like rodents, quail will react to novel stimuli including novel spatial locations by approaching the novel stimulus, even when novelty is relative (i.e., the novel stimulus has been seen but fewer times or longer ago). It is worth noting, however, that during the test trial of YMD, the novel arm has not been seen for 24 hours. At this delay, quail also do not selectively freeze to a previously fear-associated context, suggesting that 24 hours is beyond the spatial memory capacity of quail.

The observation of selective freezing at shorter intervals during CFC training shows that, like mammals, quail can associate a noxious stimulus with the context in which it was presented, leading to quail selectively freezing in the stimulus-associated context. The observation that this association is preserved following either rHp or cHp lesions is inconsistent with the majority of data on the mammalian vHp. Several conclusions are possible given this observation. It is possible that the caudal lesions made here spared enough tissue to mediate CFC. In fact, some data suggest that the functional equivalent of the vHp may be considerably larger in a bird than a mammal. Anatomical studies in pigeons [26] report that input from the nucleus taeniae of the amygdala is absent in the rostral third of the HF, but widespread in the caudal two-thirds of this region. These widespread connections suggest that the majority of the avian Hp is homologous to the ventral mammalian Hp, and the cHp lesions conducted here were not sufficient to remove this distributed structure in its entirety. This may be particularly true for Japanese quail (or Galliformes in general) relative to other avian orders. This suggestion is consistent with observations of species differences in spatial information processing. Bird species vary considerably as to the extent of the high-resolution spatially-tuned dHp analogue [29]. It would be intuitive, then, to hypothesize that birds with a smaller rHp (e.g., birds that do not cache food) would have a proportionately larger cHp. In fact, recent physiological data showing a lack of place cells in quail even in relatively rostral coordinates further suggests that the rHp may be exceptionally small in quail, even among non-food-caching birds. By extension, cHp may be exceptionally large in these birds. This proposal is consistent with a recent MRI-based quail atlas that supports the presence of a large cHp [42]. Functionally, the idea that quail may have an exceptionally large “emotional processing” centre is also consistent with the use of quail as a model of stress reactivity [43, 44]. Finally, it remains possible that CFC is entirely Hp-independent in birds. There is data in rodents implicating the prefrontal cortex in mediating CFC (reviewed by [45]). The NCL, the equivalent structure in birds, also plays a role in processing context [46, 47]. It is possible that in birds, unlike mammals, the NCL is capable of mediating CFC even in the face of extensive damage to the hippocampus. This possibility seems unlikely, however, given the extensive homology found between the avian and mammalian hippocampus to date. This is made even more unlikely by two additional observations. The first is that, even in mammals, the extent to which the dHp or vHp are selectively required for CFC is effected by relatively subtle changes in protocol [48]. Notably, the presence of a discrete cue that signals the presence of shock during training seems to make the subsequent freezing when exposed to only the context depend more on the vHp. This cue was absent in the current experiment. Additionally, at least one experiment has reported preserved CFC in rodents with selective lesions to either the dHp or vHp [12]. Importantly, in this study removal of the entire Hp dramatically reduced freezing in a shock-associated context, despite the fact that selective lesions to either half of the Hp left CFC intact. Collectively, these observations make it far more likely that, like mammals under some conditions, CFC in quail can be supported by either the rHp or cHp alone.

Despite remaining open questions concerning the extent and functional heterogeneity of the cHp, the current results demonstrate that, like its mammalian homologue, the avian Hp is functionally heterogeneous, with its rostral portion specialized for computations that support spatial learning and memory.
